# The effect of amifostine on differentiation of the human megakaryoblastic Dami cell line

**DOI:** 10.1002/cam4.759

**Published:** 2016-05-26

**Authors:** Hai‐tao Wang, Bo Yang, Bo Hu, Xiao‐hua Chi, Long‐long Luo, Hong‐qi Yang, Xiao‐ling Lang, Jing Geng, Chun‐xia Qiao, Yan Li, Xiao‐Xiong Wu, Hong‐li Zhu, Ming Lv, Xue‐chun Lu

**Affiliations:** ^1^ Department of Geriatric HematologyChinese PLA General HospitalBeijing100853China; ^2^ Institute of Basic Medical SciencesAcademy of Military Medical SciencesBeijing100039China; ^3^ Department of HematologyFirst Affiliated Hospital of Chinese PLA General HospitalBeijing100048China; ^4^ Department of Thoracic Surgery IIPeking University Cancer Hospital & InstituteBeijing100142China; ^5^ Department of PharmacyChinese PLA Rocket General HospitalBeijing100800China

**Keywords:** Amifostine, CD41a, Dami cells, DNA ploidy, transcription factor

## Abstract

Amifostine is a cytoprotective drug that was initially used to control and treat nuclear radiation injury and is currently used to provide organ protection in cancer patients receiving chemotherapy. Clinical studies have also found that amifostine has some efficacy in the treatment of cytopenia caused by conditions such as myelodysplastic syndrome and immune thrombocytopenia, both of which involve megakaryocyte maturation defects. We hypothesized that amifostine induced the differentiation of megakaryocytes and investigated this by exposing the human Dami megakaryocyte leukemia cell line to amifostine (1 mmol/L). After 12 days of amifostine exposure, optical microscopy showed that the proportion of Dami cells with diameters >20 *μ*m had increased to 24.63%. Transmission electron microscopy identified the development of a platelet demarcation membrane system, while flow cytometry detected increased CD41a expression and decreased CD33 expression on the Dami cell surface. Ploidy analysis found that the number of polyploid cells with >4N DNA content increased to 27.96%. We did not detect any elevation in the mRNA or protein levels of megakaryocytic differentiation‐associated transcription factors GATA‐binding factor 1 (GATA‐1) and nuclear factor, erythroid 2 (NF‐E2), but nuclear import assay revealed an increased nuclear translocation of these proteins. These findings indicate that amifostine induced the differentiation of Dami cells into mature megakaryocytes via a mechanism involving increased nuclear translocation of the transcription factors, NF‐E2 and GATA‐1.

## Introduction

Amifostine (WR‐2721; S‐2[3‐aminopropylamino]‐ethyl‐ phosphorothioic acid) was developed by the US Walter Reed Army Institute of Research in the 1960s for protection against nuclear radiation damage during the Cold War. Amifostine was subsequently used as a cytoprotective agent to reduce the toxicities of alkylating agents and cisplatin 1. Amifostine is known to reduce the toxic effects of chemotherapy on the kidney, bone marrow, mucous membrane, ear, and nervous system 2, 3, 4. These studies reported that amifostine did not reduce the effects of chemotherapy on tumor cells, while preventing damage to other organs in cancer patients.

Amifostine can reduce apoptosis and increase the colony‐forming ability of normal hematopoietic progenitor cells, effects that may be related to the activation of nuclear factor kappa B 5, 6. Amifostine also induces p53‐independent apoptosis in leukemia cells and inhibits their proliferation by arresting the cell cycle at the G0/G1 phase 7, 8. Amifostine produces different effects on tumor cells and normal cells because this prodrug is only activated when dephosphorylated by the cell membrane protein, alkaline phosphatase; this generates the free thiol (WR‐1065) 9. In contrast, the hypoxic conditions in tumor tissues significantly reduce amifostine uptake, as compared with normal tissues. This results in a higher drug concentration in normal tissues than in tumor tissues, producing different effects on cells 10.

Clinical studies 11, 12, 13 have also found that amifostine has some efficacy in cytopenia, including myelodysplastic syndrome (MDS) and immune thrombocytopenia (ITP). A phase I/II clinical trial conducted by List et al 13. treated 18 MDS patients with 100, 200, 400, or 740 mg/m^2^ amifostine and found that 83% of the patients who received 100–400 mg/m^2^ had improved blood counts, and that six out of 14 thrombocytopenic patients showed a 50% increase in platelet counts, as compared to their counts prior to treatment. No treatment‐related disappearance of abnormal karyotypes was found in any of these patients and only two patients showed a higher proportion of normal karyotypes, indicating that amifostine did not alter the number of abnormal clones. ITP is a disease characterized by defects in the differentiation and maturation of megakaryocytes. Fan et al. 14employed amifostine to treat 24 patients with ITP and found that all patients showed varying degrees of elevation in their platelet counts.

Megakaryocyte dysplasia or defects in megakaryocyte differentiation and maturation are found in both ITP and MDS. The amifostine‐induced increases in platelet counts in these patients cannot be explained by the classical alkaline phosphatase pathway and we speculated that amifostine might promote the differentiation and maturation of megakaryocytes. In order to investigate this, this study exposed the human megakaryocytic leukemia Dami cell line to amifostine for 12 days. First, we determined the optimal concentration of amifostine for the promotion of Dami cell differentiation. Then, the effects of amifostine on Dami cell morphology, CD41a expression, and ploidy were investigated. The results of these investigations demonstrated that the differentiation‐promoting effect of amifostine involved altered nuclear translocation of the transcription factors, GATA‐binding factor 1 (GATA‐1) and nuclear factor, erythroid 2 (NF‐E2).

## Methods

### Reagents

Amifostine was granted by Dalian Joymeo Pharmaceutical Co., Ltd, stored in the dark at 4°C, and dissolved prior to use. Mouse anti‐human CD41a‐PE antibody, rabbit anti‐human NF‐E2 antibody, Giemsa stain solution, and propidium iodide (PI) staining solution were purchased from Santa Cruz Biotechnology, Inc. (USA). Rabbit anti‐human GATA‐1 antibody was purchased from CST, Inc. (USA). Horseradish peroxidase‐labeled goat anti‐rabbit/goat anti‐mouse secondary antibodies were purchased from Zhongshan Golden Bridge Co., Ltd. (Beijing, China). SYBR^®^ Green Real‐time PCR Master Mix was purchased from the Takara Bio, Inc. (Tokyo, Japan) and the reverse transcription kit was purchased from TransGen Biotech Co., Ltd. (Beijing, China), and DAPI dye was purchased from Pierce.

### Cell culture and proliferation

The Dami cell line was purchased from the American Type Culture Collection (USA). These cells were cultured in RPMI1640 (Hyclone, USA) medium containing 10% heat‐inactivated fetal bovine serum (FBS) and 100 U/mL double antibiotics in suspension at 37°C, with 5% CO_2_ and saturated humidity. In this experiment, the Dami cells were cultured in 6‐well plates (Corning, Inc., Tewksbury, MA, USA) to a cell density of 5 × 10^5^ cells/mL and passaged once every 2 days. The final concentration of amifostine in the medium was adjusted to 1 mmol/L. The Dami cells were counted every morning at 10:00 am using the hemocytometer, in order to construct growth curves.

### Cell analyses

#### Light microscopy

The density of Dami cells treated with amifostine for 0 (Control), 4, 8, or 12 days was adjusted to 1 × 10^6^ cells/mL, and 1 mL of the cell suspension was transferred into each well of a 24‐well plate. Cell morphology was observed and imaged using a light microscope. In addition, 1 × 10^6^ cells were harvested and resuspended after being washed once with phosphate‐buffered saline (PBS). This suspension (150 *μ*L) was deposited on slides using a cytocentrifuge, followed by Giemsa staining for 20 min. After being rinsed with pure water, these slides were observed under the light microscope.

#### Transmission electron microscopy

A total of 2 × 10^6^ Dami cells were harvested after 12‐day exposure to amifostine and fixed in phosphate buffer (0.1 mol/L, pH 7.2) containing 5% glutaraldehyde (Sigma Chemical Co., St Louis, MO, USA). The cells were washed once, fixed with osmium tetroxide, and embedded in epoxy resin. The mounted cells were then sectioned and stained with uranyl acetate and lead citrate prior to observation using a transmission electron microscope.

### Flow cytometry

#### Cell surface expression of CD41a

The expression of CD41a on the Dami cell surface was determined after 0 (Control), 4, 8, or 12 days of treatment with amifostine. At day 12, the levels of cell surface CD33 and CD34 were also determined. About 1 × 10^6^ cells were collected in 1.5‐mL tubes and washed once with a stream of prechilled (4°C) washing buffer. The indicated antibody (100 *μ*L) was added and evenly mixed by pipetting. The cells were then incubated in the dark at 37°C for 30 min, washed three times with washing buffer, and analyzed by flow cytometry.

#### DNA ploidy determination

After 0, 4, 8, or 12 days of treatment with amifostine, 2 × 10^6^ cells were harvested and washed once with prechilled PBS (−20°C) containing 2.5% FBS and 0.5% NaN_3_. After the addition of 75% ethanol, the cell suspension was evenly mixed by gentle pipetting and fixed at −20°C for at least 24 h. The cells were then washed once with PBS and 100 *μ*L RNase was added to achieve a final concentration of 1 mg/mL, followed by incubation in the dark at 37°C for 30 min. PI dye (100 *μ*L) was added to achieve a final concentration of 0.1 mg/mL, and incubated in the dark at room temperature for 5 min, followed by flow cytometric analysis. DNA ploidy was analyzed using the CellQuest Pro software.

### Determination of transcription factor mRNA levels

After 0, 4, 8, or 12 days of treatment with amifostine, 1 × 10^6^ cells were harvested and total RNA was extracted using Trizol reagent. The extracted RNA (1 *μ*g) was reverse transcribed to cDNA using a reverse transcription kit (TransGen Biotech Co., Ltd.). Real‐time quantitative PCR was performed using an iCycler thermocycler (Bio‐Rad, Hercules, CA, USA) platform with a melting temperature of 64°C. The expression levels of the target genes (aNF‐E2, fNF‐E2, and GATA‐1) were calculated relative to that of a housekeeping gene, glyceraldehyde 3‐phosphate dehydrogenase (GAPDH). The results were analyzed using Bio‐Rad iQ5 standard edition software.

### Determination of transcription factor protein levels

After 0, 4, 8, or 12 days of treatment with amifostine, 1 × 10^6^ Dami cells were harvested and lysed by incubation with M2 buffer at 0°C for 15 min, followed by centrifugation at 12,000 g for 15 min. The extracted protein samples were separated by sodium dodecyl sulfate‐polyacrylamide gel electrophoresis (12%), transferred to a nitrocellulose membrane, and incubated overnight at 4°C with rabbit anti‐human NF‐E2 or GATA‐1, or mouse anti‐human GAPDH antibodies. The membrane was then blocked using 5% skim milk for 1 h at room temperature prior to incubation with horseradish peroxidase‐labeled goat anti‐rabbit of goat anti‐mouse secondary antibodies, as appropriate, at room temperature for 1 h, followed by detection using enhanced chemiluminescence in a darkroom (Cell Signaling, Boston, MA, USA).

### Determination of transcription factor nuclear translocation

After 12 days of treatment with amifostine, Dami cells were deposited onto slides, incubated with GATA‐1/NF‐E2 antibodies at room temperature for 30 min, washed three times with PBS, fixed with 4% paraformaldehyde for 15 min, washed three times with PBS, and stained with 50 *μ*g/mL DAPI dye in the dark at room temperature for 5 min. After that, the cells were washed three times with PBS prior to visualization using laser confocal microscopy. LSM Image Browser software was used to process these images.

### Statistical analysis

The SPSS 13.0 software package was used for data analysis. The data were presented as the mean ± the standard deviation and the study groups were compared using the *t*‐test. Treatment‐associated differences in the proportion of cells with a diameter >20 *μ*m, and in the expression of CD41a and CD33 were analyzed using the Wilcoxon test. *P* < 0.05 was considered statistically significant.

## Results

### Determination of the optimal concentration of amifostine for inducing Dami cell differentiation

**Figure 1:**
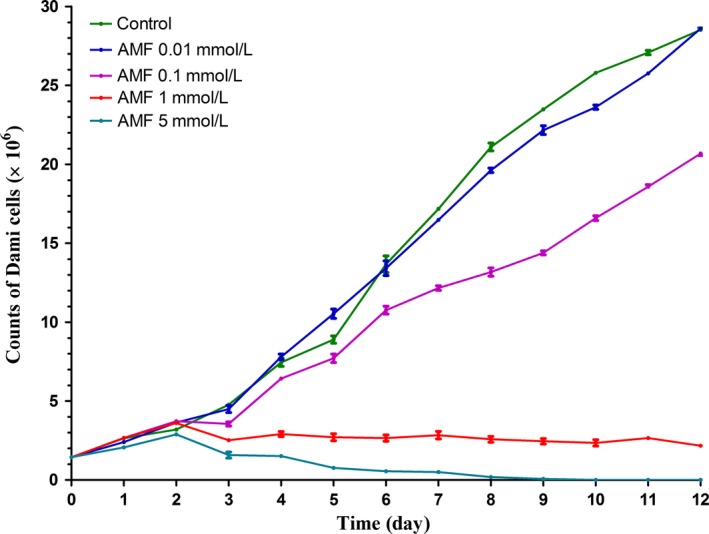
Dami cell growth curves in the presence of the indicated concentrations of amifostine. Amifostine (0.1 – 5 mmol/L) inhibited the proliferation of Dami cells. The number of Dami cells remained constant during exposure to 1 mmol/L amifostine, indicating cell differentiation.

**Figure 2:**
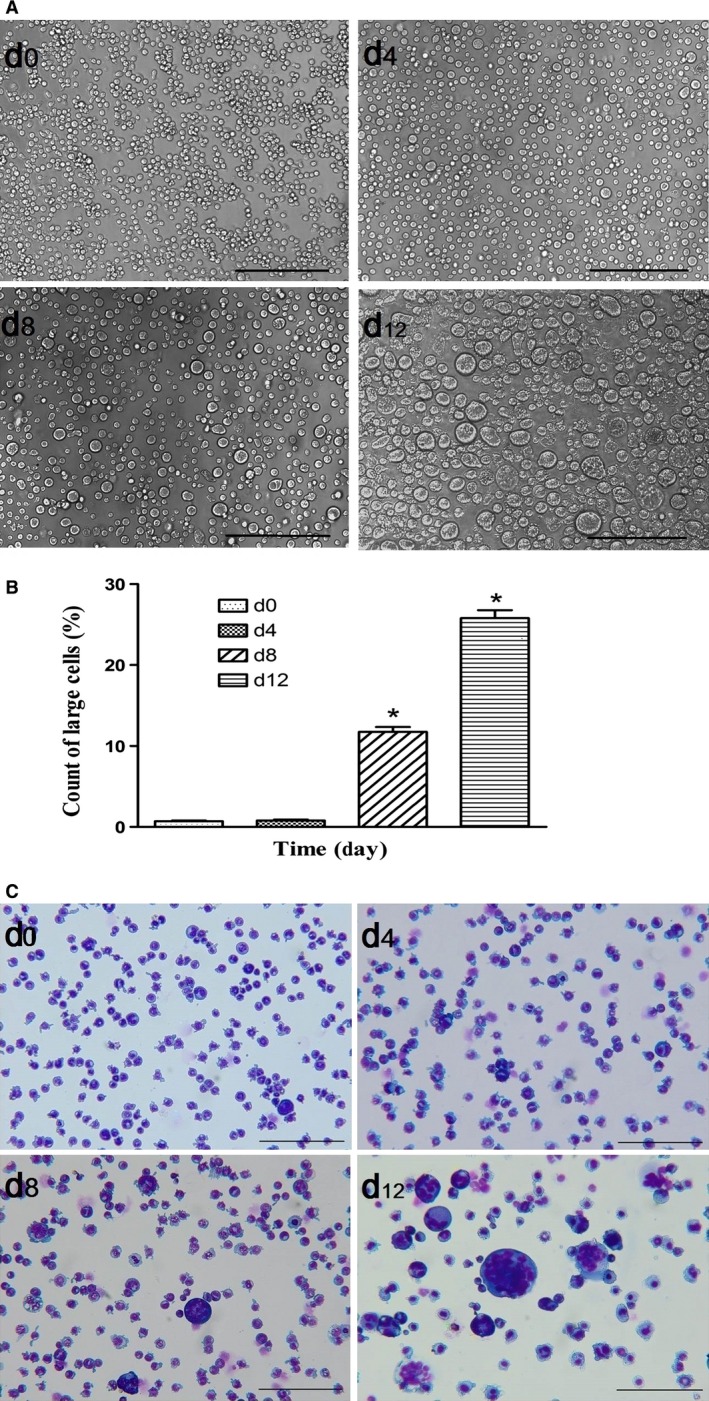
The morphology of Dami cells exposed to 1 mmol/L amifostine for the indicated number of days (d). The Dami cell volume and cytoplasm increased over time (A) and the proportion of cells with diameter >20 *μ*m was counted in three randomly selected fields (B). The differences between the control cells and cells treated with amifostine for 4 and 8 days were statistically significant (*P* < 0.05). Giemsa staining (C) confirmed that the differentiated Dami cells had increased cytoplasmic volume and DNA ploidy. Cells with 32N ploidy were also observed.

**Figure 3:**
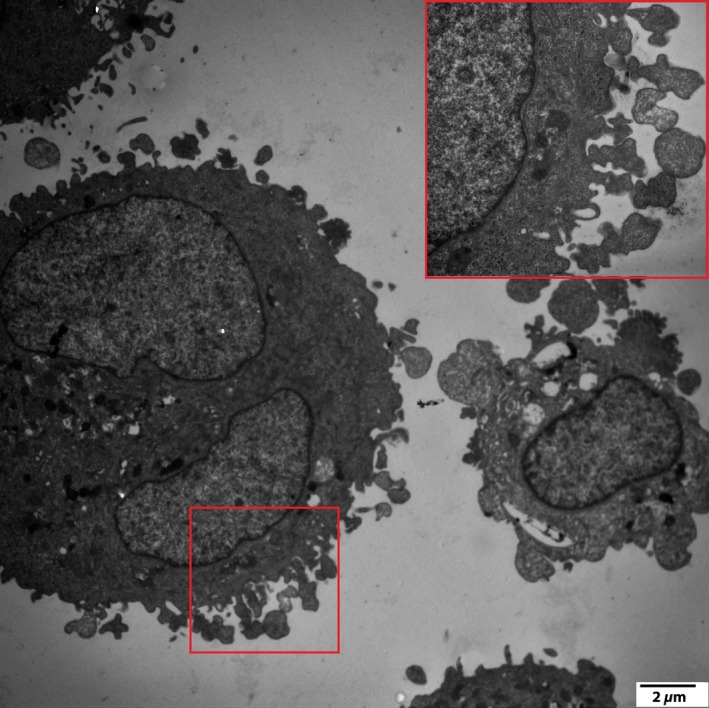
Transmission electron microscopy image of Dami cells treated with 1 mmol/L amifostine for 12 days. Mature megakaryocyte features are visible, including well‐developed ribosomes and endoplasmic reticulum, and sponge‐like edges indicating the platelet demarcation membrane system.

Based on the concentrations of amifostine previously reported to induce cell differentiation 5, 8, 15, we exposed Dami cells to 0.01, 0.1, 1, or 5 mmol/L amifostine. The cells were treated for up to 12 days and the number of Dami cells was counted in order to construct growth curves (Fig. 1). Under the light microscope, cells in the control group appeared to be spherical, with diameters of 10–15 *μ*m. We found that >1 mmol/L amifostine significantly inhibited the proliferation of Dami cells, as compared with the control cells (*P *= 0.0097). It is noteworthy that in the presence of 1 mmol/L amifostine, the number of Dami cells remained constant for the duration of the treatment, but the cell volume increased over time (Fig. 2A), indicating cell differentiation. A previous report 16 indicated that the Dami cell diameter increased to >20 *μ*m during their differentiation into megakaryocytes. We therefore, randomly selected three microscope fields and calculated the proportion of cells with a diameter >20 *μ*m. As the amifostine treatment duration increased, the proportion of cells with a diameter >20 *μ*m gradually increased and some cells were even >100 *μ*m in diameter (Fig. 2A); this analysis identified a statistically significant difference between amifostine‐treated and control cells (Fig. 2B). In order to explore the effect of amifostine on differentiation further, we performed morphological investigations of Dami cells exposed to amifostine for 12 days. This included Giemsa nuclear staining and transmission electron microscopy to observe cellular ultrastructure. Giemsa staining identified significantly lower nucleus‐to‐cytoplasm volume ratios in the amifostine‐treated cells and an increased number of nuclei (Fig. 2C). Electron microscopy revealed that in comparison to the amifostine‐treated cells, control Dami cells exhibited a higher nucleus‐to‐cytoplasm volume ratio, higher numbers of nucleoli 4, 5, the presence of nuclear euchromatin, and abundant cytoplasmic ribosomes. In contrast, the amifostine‐treated cells showed larger diameters of up to about 100 *μ*m with irregular and sponge‐like cell edges and platelet demarcation membrane systems (Fig. 3).

### Characteristics of the mature Dami cells after amifostine‐induced differentiation

**Figure 4:**
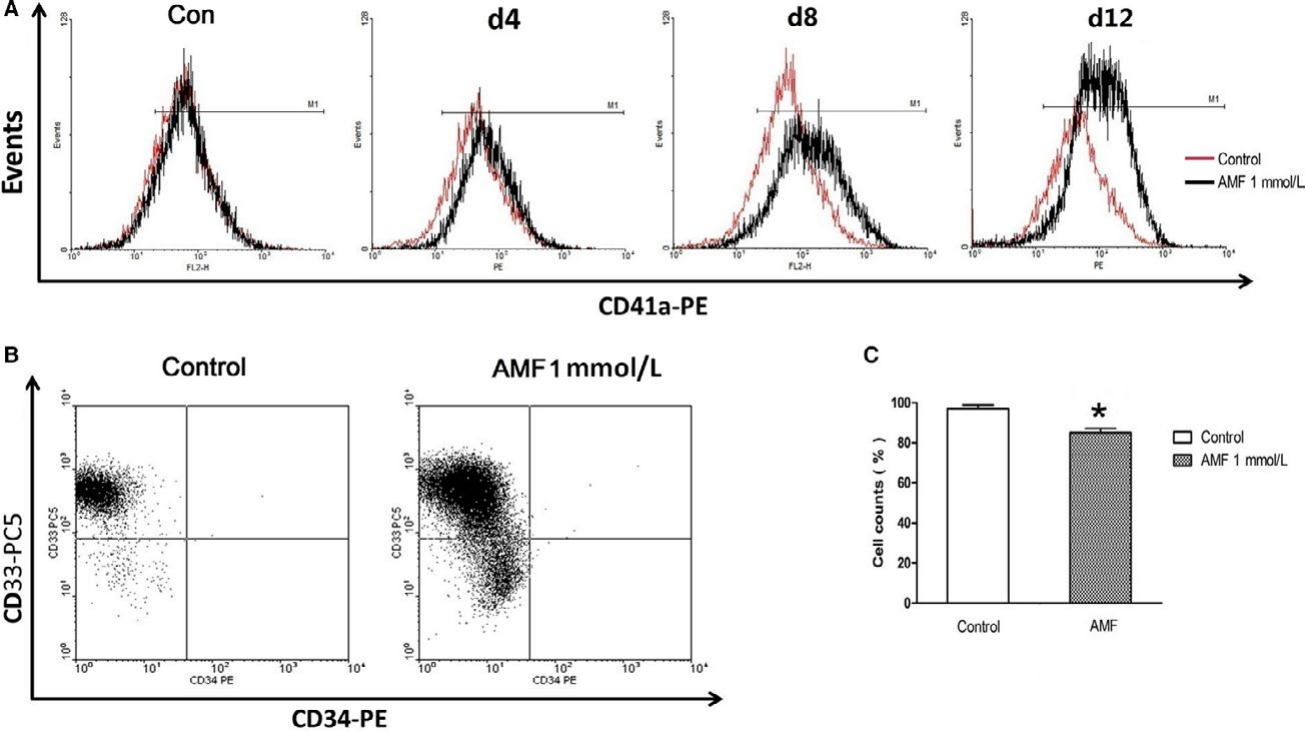
Flow cytometric analysis of Dami cell surface CD41a, CD33, and CD34 expression during amifostine treatment (1 mmol/L) for the indicated times. Increased expression of CD41a was observed (A). Dami cells are megakaryoblasts, and did not express CD34 (B). After 12‐day exposure to amifostine induction, cell surface CD33 expression was reduced (C).

Dami cells are megakaryoblasts, expressing cell surface megakaryocyte‐specific antigens including CD41a (GpIIb/IIIa complex) and CD61a(Ib), as well as myeloid antigens such as CD33 17, 18. During the maturation of megakaryocytes, the expression of CD41a gradually increases while the expression of CD33 gradually decreases. We examined the expression of CD41a, CD33, and CD34 on the surface of Dami cells exposed to amifostine. This study revealed very weak expression of CD34 and found that as the treatment duration increased, the expression of CD41a increased and that of myeloid antigen CD33 decreased (Fig. 4B).

**Figure 5:**
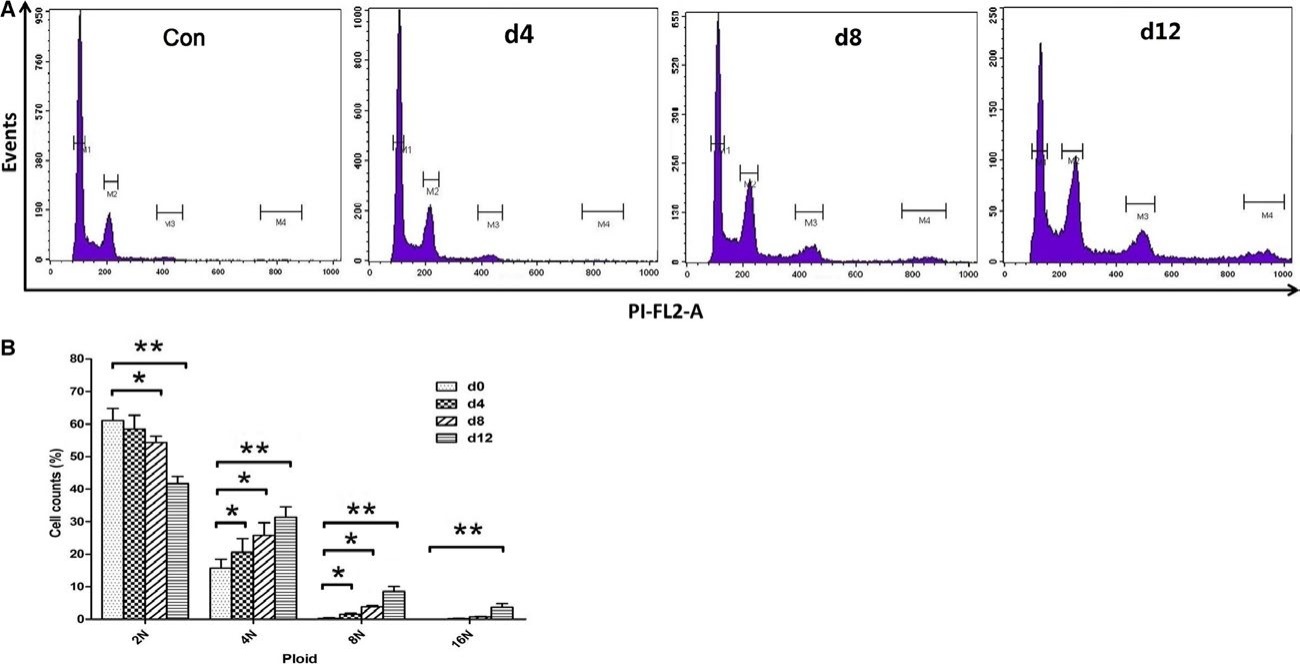
DNA ploidy in Dami cells exposed to 1 mmol/L amifostine for the indicated number of days (d). The proportion of 2N cells decreased, while the proportion of 4N and 8N cells increased, as the treatment duration increased; 16N cells were also observed. The differences between the control cells (A) and those treated with amifostine were statistically significant (B). *P < 0.05; **P < 0.01.

In addition, DNA ploidy is also an important marker of megakaryocyte differentiation and maturation. DNA ploidy gradually increases as megakaryocytes mature 19. In this study, control cells (day 0 of treatment) were mostly 2N (62.46%) or 4N (15.63%), while less than 1% were 8N or 16N. After 12 days of amifostine treatment, the proportion of 8N cells was significantly increased to 8.83% and 16N cells were also observed; these accounted for 3.43% of the total cell number. The differences between the control and treatment groups were statistically significant (*P* < 0.05; Fig. 5).

### Amifostine‐induced changes in Dami cell transcription factor expression

**Figure 6:**
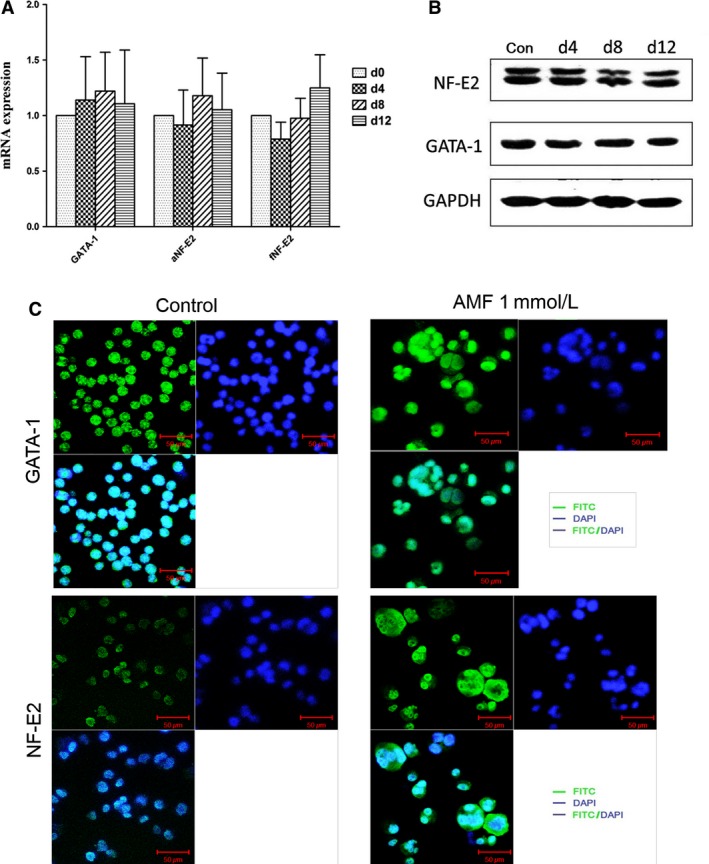
The effects of amifostine exposure (1 mmol/L) for the indicated numbers of days (d) on the differentiation‐related transcription factors, GATA‐binding factor 1 (GATA‐1) and nuclear factor, erythroid 2 (NF‐E2), in Dami cells. Amifostine did not alter the mRNA (A) or protein (B) expression levels of GATA‐1 and NF‐E2. GAPDH, glyceraldehyde 3‐phosphate dehydrogenase. After 12 days of treatment with amifostine, the nuclear translocation of GATA‐1 and NF‐E2 were enhanced in Dami cells (C).

The later stages of megakaryocyte maturation include mitosis, cytoplasmic maturation, and the formation of thromboblasts. These processes require the concerted effects of various transcription factors, which control the maturation of megakaryocytes to eventually produce platelets. In this study, the mRNA levels of GATA‐1, aNF‐E2, or fNF‐E2 showed no significant differences in the amifostine‐treated cells, as compared to controls (Fig. 6A, *P* > 0.05). In addition, examination of the protein levels of these transcription factors did not identify any significant changes over time (Fig. 6B). However, even when the overall transcription factor levels remain unchanged, their activation and deactivation can regulate gene transcription. To investigate this, we performed nuclear import assays and found accumulation of GATA‐1 and NF‐E2 in the Dami cell nuclei after 12‐day exposure to amifostine (Fig. 6C). By western blotting analysis of GATA‐1 and NF‐E2 in nuclear and cytosolic in the Dami cell, we found that the express level of both GATA‐1 and NF‐E2 increased in nuclear, while in cytosolic not after 12‐day treatment of amifostine (Fig. S1). This indicated that amifostine promoted Dami cell differentiation by increasing transcription factor translocation from the cytoplasm into the nucleus.

## Discussion

The cell differentiation‐promoting effects of amifostine were first reported by Kashiwakura et al. 20, who used amifostine in combination with thrombopoietin (TPO) to induce in vitro differentiation of CD34+ cells from placental and umbilical cord blood into megakaryocytes. They discovered that the number of megakaryocyte progenitor cells increased by 83‐fold in the presence of amifostine, as compared with a TPO‐treated group. However, this combined study of amifostine and TPO did not identify the direct effect attributable to amifostine on the differentiation of hematopoietic stem cells. We therefore, aimed to clarify the role of amifostine using the Dami cell line, which provides a well‐developed research tool to study megakaryocyte differentiation 21, 22.

Hematopoietic stem cells differentiate to form mature megakaryocytes via many stages, including megakaryocyte/erythroid progenitor cells, megakaryocyte progenitor cells, megakaryoblasts, promegakaryocytes, and granular megakaryocytes. This maturation process is accompanied by an increase in cell volume and chromosomal ploidy. The chromosomal ploidy of megakaryoblasts is 2N and the average cell diameter is about 20 *μ*m. At the promegakaryocyte stage, polyploid cells start to emerge and this can reach 8N, while the cell diameter increases to about 30 *μ*m. The ploidy of mature megakaryocytes (granular or platelet‐producing) is usually >32N, with a large cell volume of >40 *μ*m and the presence of the platelet demarcation membrane system 16, 23. In this study, the control megakaryoblast Dami cells had a diameter range of 10–15 *μ*m. This diameter and chromosomal ploidy increased over time during exposure to amifostine, with cells >20 *μ*m and 4N observed after 4 days. After treatment with amifostine for 12 days, the proportion of cells with a diameter >20 *μ*m was 24.83%, and 4N cells accounted for 43.82% of the total cell number. In addition, transmission electron microscopy revealed the presence of a platelet demarcation membrane system, suggesting that these cells had differentiated into mature megakaryocytes.

The megakaryocyte immunophenotype also changes during the maturation process. Megakaryocyte/erythroid progenitor cells have an immunophenotype of Lin^−^CD34^+^CD33^+^D38^+^IL3R*α*−CD45RA− 24. During differentiation into mature megakaryocytes, the expression of CD33 and CD34 on the cell surface decreases, while the expression of CD41a, CD42b, CD45, and CDw32 increases 23, 24, 25. CD41a is a characteristic molecular marker of megakaryocyte differentiation and maturation 18. In this study, flow cytometry confirmed that the Dami cell surface CD41a expression gradually increased with the treatment duration and that the expression of CD33 decreased, consistent with the megakaryocyte differentiation process.

The mechanism involved in amifostine‐induced differentiation of Dami cells remains unclear. It has been reported 21 that the protein expression levels of the transcription factors, GATA‐1, Fli‐1, and NF‐E2, increased during the differentiation of Dami cells induced by fluorinated esters and TPO. Fli‐1 is involved in controlling the mitosis and cytoplasmic maturation of megakaryocytes, NF‐E2 (including its splice isoforms, aNF‐E2 and fNF‐E2) is involved in the formation of platelets, while GATA‐1 plays an important role in all three of these processes 26, 27, 28, 29. We hypothesized that amifostine increased the expression of these transcription factors. However, we did not detect elevated mRNA or protein levels of GATA‐1, Fli‐1, or NF‐E2. Previous studies have shown that transcription can be activated by increased activities of transcription factors in the absence of changes in their expression. Our nuclear import assay showed that amifostine activated nuclear translocation of GATA‐1 and NF‐E2. This result indicated that amifostine induced the differentiation of Dami cells by modulating transcription factor activities, rather than by increasing their expression.

In conclusion, we found that amifostine induced the differentiation of Dami cells and that this effect was associated with increased nuclear translocation of the transcription factors, GATA‐1 and NF‐E2. Further studies should be carried out to confirm these effects of amifostine on megakaryocyte differentiation in other cell lines and in vivo.

## Conflict of Interest

The authors declare no conflict of interest.

## Supporting information


**Figure S1.** After 12 days of amifostine exposure, expression of GATA‐1 and NF‐E2 in nucleus increased, while their expression in cytosol did not increase. GATA‐binding factor 1 (GATA‐1), nuclear factor, erythroid 2 (NF‐E2), GAPDH and glyceraldehyde 3‐phosphate dehydrogenase.

## References

[1] Valeriote, F. , and S. Tolen . 1982 Protection and potentiation of nitrogen mustard cytotoxicity by WR‐2721. Cancer Res. 42:4330–4331. 6290032

[2] Capizzi, R. L. , B. J. Scheffler , and P. S. Schein . 1993 Amifostine‐mediated protection of normal bone marrow from cytotoxic chemotherapy. Cancer 72(11 Suppl.):3495–3501. 8242582 10.1002/1097-0142(19931201)72:11+<3495::aid-cncr2820721617>3.0.co;2-b

[3] Gurney, J. G. , J. K. Bass , A. Onar‐Thomas , J. Huang , M. Chintagumpala , E. Bouffet , et al. 2014 Evaluation of amifostine for protection against cisplatin‐induced serious hearing loss in children treated for average‐risk or high‐risk medulloblastoma. Neuro Oncol. 16:848–855. 24414535 10.1093/neuonc/not241 PMC4022215

[4] Kanter, M. , Y. Topcu‐Tarladacalisir , and C. Uzal . 2011 Role of amifostine on acute and late radiation nephrotoxicity: a histopathological study. In Vivo. 25:77–85. 21282738

[5] List, A. F. , R. Heaton , B. Glinsmann‐Gibson , and R. L. Capizzi . 1998 Amifostine stimulates formation of multipotent and erythroid bone marrow progenitors. Leukemia 12:1596–1602. 9766505 10.1038/sj.leu.2401151

[6] Romano, M. F. , A. Lamberti , R. Bisogni , C. Garbi , A. M. Pagnano , P. Auletta , et al. 1999 Amifostine inhibits hematopoietic progenitor cell apoptosis by activating NF‐kappaB/Rel transcription factors. Blood 94:4060–4066. 10590050

[7] Huang, X. K. , X. Z. Gao , P. Burke , A. Raza , and H. D. Preisler . 1997 The effects of amifostine on the clonogenic proliferation in vitro of myelodysplastic and acute and chronic myelogenous leukemia cells. ASCO Proc. 16:1848a.

[8] Ribizzi, I. , J. W. Darnowski , F. A. Goulette , M. R. Sertoli , and P. Calabresi . 2000 Amifostine cytotoxicity and induction of apoptosis in a human myelodysplastic cell line. Leuk. Res. 24:519–525. 10781687 10.1016/s0145-2126(00)00007-2

[9] Purdie, J. W. , E. R. Inhaber , H. Schneider , and J. L. Labelle . 1983 Interaction of cultured mammalian cells with WR‐2721 and its thiol, WR‐1065: implications for mechanisms of radioprotection. Int. J. Radiat. Biol. Relat. Stud. Phys. Chem. Med. 43:517–527. 6303973 10.1080/09553008314550611

[10] Smoluk, G. D. , R. C. Fahey , P. M. Calabro‐Jones , J. A. Aguilera , and J. F. Ward . 1988 Radioprotection of cells in culture by WR‐2721 and derivatives: form of the drug responsible for protection. Cancer Res. 48:3641–3647. 2837320

[11] Grossi, A. , A. Fabbri , V. Santini , F. Leoni , C. Nozzoli , G. Longo , et al. 2000 Amifostine in the treatment of low‐risk myelodysplastic syndromes. Haematologica 85:367–371. 10756361

[12] Schanz, J. , H. Jung , B. Wormann , W. Gassmann , T. Petersen , A. Hinke , et al. 2009 Amifostine has the potential to induce haematologic responses and decelerate disease progression in individual patients with low‐ and intermediate‐1‐risk myelodysplastic syndromes. Leuk. Res. 33:1183–1188. 19411105 10.1016/j.leukres.2009.03.027

[13] List, A. F. , F. Brasfield , R. Heaton , B. Glinsmann‐Gibson , L. Crook , R. Taetle , et al. 1997 Stimulation of hematopoiesis by amifostine in patients with myelodysplastic syndrome. Blood 90:3364–3369. 9345018

[14] Fan, H. , H. L. Zhu , S. X. Li , X. C. Lu , B. Zhai , B. Guo , et al. 2011 Efficacy of amifostine in treating patients with idiopathic thrombocytopenia purpura. Cell Biochem. Biophys. 59:7–12. 21104159 10.1007/s12013-010-9100-5

[15] Neumeister, P. , G. Jaeger , M. Eibl , S. Sormann , W. Zinke , and W. Linkesch . 2001 Amifostine in combination with erythropoietin and G‐CSF promotes multilineage hematopoiesis in patients with myelodysplastic syndrome. Leuk. Lymphoma 40(3–4):345–349. 11426556 10.3109/10428190109057933

[16] Kaushansky, K. , M. A. Lichtman , E. Beutler , T. J. Kipps , J. T. Prchal , and U. Seligsohn . 2010 pp. 1721–1722 *in* Williams Hematology 8thed. Chapter 113. Megakaryopoiesis and Thrombopoiesis. McGraw‐Hill Education. ISBN 0071621512, 9780071621519.

[17] Greenberg, S. M. , D. S. Rosenthal , T. A. Greeley , R. Tantravahi , and R. I. Handin . 1988 Characterization of a new megakaryocytic cell line: the Dami cell. Blood 72:1968–1977. 3196874

[18] Mostafa, S. S. , E. T. Papoutsakis , and W. M. Miller . 2001 Oxygen tension modulates the expression of cytokine receptors, transcription factors, and lineage‐specific markers in cultured human megakaryocytes. Exp. Hematol. 29:873–883. 11438210 10.1016/s0301-472x(01)00658-0

[19] Levine, R. F. , K. C. Hazzard , and J. D. Lamberg . 1982 The significance of megakaryocyte size. Blood 60:1122–1131. 7126866

[20] Kashiwakura, I. , M. Murakami , O. Inanami , Y. Hayase , T. A. Takahashi , M. Kuwabara , et al. 2002 Effects of amifostine on the proliferation and differentiation of megakaryocytic progenitor cells. Eur. J. Pharmacol. 437(1–2):19–25. 11864634 10.1016/s0014-2999(02)01270-0

[21] Lev, P. R. , N. P. Goette , A. C. Glembotsky , R. P. Laguens , P. M. Meckert , J. P. Salim , et al. 2011 Production of functional platelet‐like particles by the megakaryoblastic DAMI cell line provides a model for platelet biogenesis. Platelets 22:28–38. 21143024 10.3109/09537104.2010.515271

[22] Briquet‐Laugier, V. , N. El Golli , P. Nurden , C. Lavenu‐Bombled , A. Dubart‐Kupperschmitt , A. Nurden , et al. 2004 Thrombopoietin‐induced Dami cells as a model for alpha‐granule biogenesis. Platelets 15:341–344. 15370095 10.1080/09537100410001721342

[23] Tomer, A. 2004 Human marrow megakaryocytedifferentiation: multiparameter orrelative analysis identifies von Willebrand factor as a sensitive and distinctive marker for early (2N and 4N) megakaryocytes. Blood 104:2722–2727. 15198950 10.1182/blood-2004-02-0769

[24] Yu, M. , and A. B. Cantor . 2012 Megakaryopoiesis and thrombopoiesis: an update on cytokines and lineage surface markers. Methods Mol. Biol. 788:291–303. 22130715 10.1007/978-1-61779-307-3_20

[25] Qiao, X. , M. Loudovaris , K. Unverzagt , D. E. Walker , S. L. Smith , J. Martinson , et al. 1996 Immunocytochemistry and flow cytometry evaluation of human megakaryocytes in fresh samples and cultures of CD34 + cells. Cytometry 23:250–259. 8974870 10.1002/(SICI)1097-0320(19960301)23:3<250::AID-CYTO8>3.0.CO;2-M

[26] Szalai, G. , A. C. LaRue , and D. K. Watson . 2006 Molecular mechanisms of megakaryopoiesis. Cell. Mol. Life Sci. 63:2460–2476. 16909203 10.1007/s00018-006-6190-8 PMC11136211

[27] Tijssen, M. R. , and C. Ghevaert . 2013 Transcription factors in late megakaryopoiesis and related platelet disorders. J. Thromb. Haemost. 11:593–604. 23311859 10.1111/jth.12131 PMC3824237

[28] Muntean, A. G. , L. Pang , M. Poncz , S. F. Dowdy , G. A. Blobel , and J. D. Crispino . 2007 Cyclin D‐Cdk4 is regulated by GATA‐1 and required for megakaryocyte growth and polyploidization. Blood 109:5199–5207. 17317855 10.1182/blood-2006-11-059378 PMC1890844

[29] Lecine, P. , J. L. Villeval , P. Vyas , B. Swencki , Y. Xu , and R. A. Shivdasani . 1998 Mice lacking transcription factor NF‐E2 provide in vivo validation of the proplatelet model of thrombocytopoiesis and show a platelet production defect that is intrinsic to megakaryocytes. Blood 92:1608–1616. 9716588

