# Supplementary figures and images for: The effect of amifostine on differentiation of the human megakaryoblastic Dami cell line
Source: Cancer Med. 2016 May 26;5(8):2012–21. doi: 10.1002/cam4.759 (PMC4884634; doi:10.1002/cam4.759)

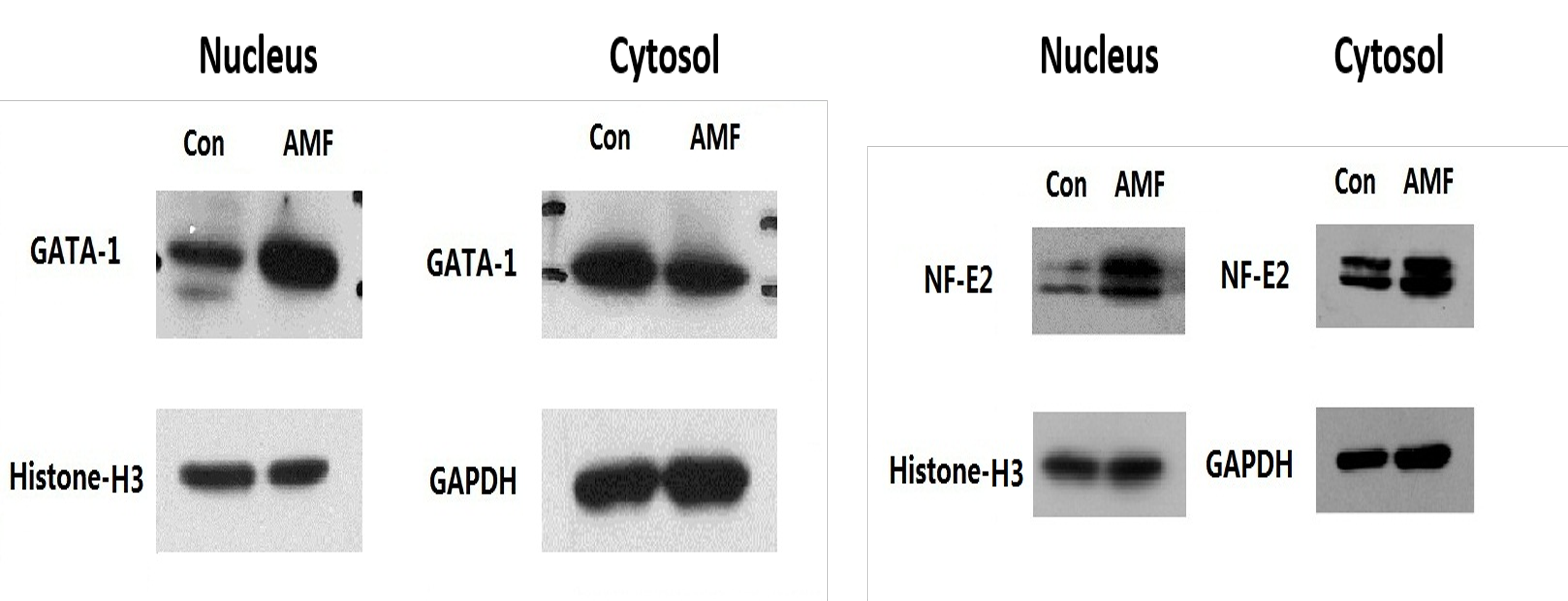

Supplement: Supplementary file 1 — Figure S1. After 12 days of amifostine exposure, expression of GATA‐1 and NF‐E2 in nucleus increased, while their expression in cytosol did not increase. GATA‐binding factor 1 (GATA‐1), nuclear factor, erythroid 2 (NF‐E2), GAPDH and glyceraldehyde 3‐phosphate dehydrogenase. [file CAM4-5-2012-s001.tif]
